# Evaluation of educational quality performance on virtual campuses using fuzzy inference systems

**DOI:** 10.1371/journal.pone.0232802

**Published:** 2020-05-29

**Authors:** Antonio Cervero, Adrián Castro-Lopez, Lucía Álvarez-Blanco, María Esteban, Ana Bernardo

**Affiliations:** 1 Department of Psychology, University of Oviedo, Oviedo, Spain; 2 Department of Business Administration, University of Oviedo, Oviedo, Spain; 3 Department of Educational Sciences, University of Oviedo, Oviedo, Spain; Hefei University of Technology, CHINA

## Abstract

The general objective of this study is to analyze student satisfaction with the use of virtual campuses in university teaching in order to discover the main variables influencing the overall online teaching-learning process that give quality to the virtual educational process. To this end, an *ex-post-facto* research methodology was applied to 1084 university students, who completed an *ad hoc* designed questionnaire, which allowed us to carry out descriptive analysis, classification trees and fuzzy inference systems using SPSS and Matlab software. The results suggest that four variables predominantly influence the quality of the teaching-learning processes in virtual campuses: satisfactory teacher responses to student questions and observations, the positive attitude of teachers towards the use of information and communication technologies, students having appropriate digital skills, and activities that encourage ideas and debate.

## Introduction

The arrival of the “*knowledge society*” brings with it enormous changes in the spheres of education, science and technology, not only for the complexity of the processes by which knowledge and information circulates, but also for the institutional and work-related changes it has generated. These changes, which are particularly significant in institutions like universities, tend to be most present in the creation and transmission of knowledge [1] and have led universities to undergo internal reorganizations which have resulted in the creation of the European Higher Education Area (EHEA).

What stands out in this context is the interest in the continual improvement of the quality of educational processes in universities. There is a problem with that concept, as there does not seem to be an operational consensus of what it specifies. Despite that, there is broad agreement on the existence of some factors that should be considered when looking at this idea, factors such as the evaluation of teachers, content, the learning environment, the combination of theory and practice, and particularly the satisfaction of end-users, the students, with the overall process [2].

Various models with varying numbers of dimensions have appeared, with those models focused on student satisfaction with the educational process being particularly interesting [3]. Currently, in those models, a key role is played by the integration of information and communication technologies (ICT) for didactic purposes because, in addition to other aspects, they change how we communicate, how we relate to content and how we create and share information [4].

Universities have been integrating virtual campuses into their structures through the creation of virtual learning environments and platforms [5] which work as packages containing everything necessary to implement technological modules and tools, and are occasionally stand-alone. The most commonly used at university level is Moodle, which has given rise to a real concept of the digital university [6].

The evaluation of the teaching-learning process in virtual campuses must consider the resources used, their aims and information-accessibility, without forgetting that the analysis of an educational process goes beyond the campus and its instruments, but rather includes a more pedagogical side which considers the process of teacher planning, communication interactions and abilities to use the technological tools [7]. Similarly, it is essential to understand the student perspective. Both the principles of online teaching and the foundations of the EHEA make particular reference to translating the emphasis of the teaching process to the learning process. The student assumes the protagonist’s role, which aims to respect individuality in the acquisition of competences [8]. This brings with it profound changes to the role of the teacher, which changes from being centered on the transmission of knowledge to being more of a guide and facilitator in the learning process [9].

It is worth reiterating that there are many studies looking at students’ perceptions of the use of technological platforms and online educational experiences in a holistic way, including projects which have tried to validate quality analysis models of campuses at the European level, such as Excellence+ and First Dual Mode Distance Learning Benchmarking Club [10]. Others have focused more on specific aspects of the educational process [11], such as the way that the use of virtual campuses can condition the educational process [12]. However, and despite the above, few studies have combined student perception with the assessment of educational quality based on the pedagogical and didactic planning criteria implemented by university teachers [13], this aspect being of fundamental importance as it allows for the indirect establishment of a criterion of satisfaction of the final user.

With all that in mind, this study aims to analyze the level of student satisfaction with the use of virtual campuses in university teaching, determining the main variables of the online teaching-learning process which, according to students, give it quality. To that end we have used a relatively novel, rigorous, reliable variety of analysis applied to the educational sphere, using techniques based on artificial intelligence such as fuzzy inference systems, which can effectively manage the uncertainty associated with human action and behavior [14].

## Materials and methods

With regard to the ethical aspects of the study, the authors state that it could not be reviewed and approved by an institutional review board (ethics committee) before starting the study, given that the University of Oviedo did not have such a committee until 2019, when it began operating, the data being collected earlier. However, in the request for information, special importance was given to this aspect by following the international and institutional protocols related to scientific research, informing the subjects about the objective of the study, data protection and guarantees of anonymity and confidentiality; and obtaining their informed consent for the completion of the questionnaire and the exploitation of results for scientific research purposes only. This section describes the methodological framework used in this work.

### Population and sample

The population of this study are students in the Spanish public universities who use the virtual campus regularly in their courses. The sample analysed was made up of 1084 students, the majority of whom were attending courses at a public university in the north of Spain (78.6%) that has its own virtual campus. The other part of the sample was made up of distance-learning students at a public university (15.9%), and a smaller subgroup attended courses in different universities (5.5%). The sample represents a student body attending various higher education institutions, both private and public and both attending classes physically, and distance/online-learning.

**Fig 1:**
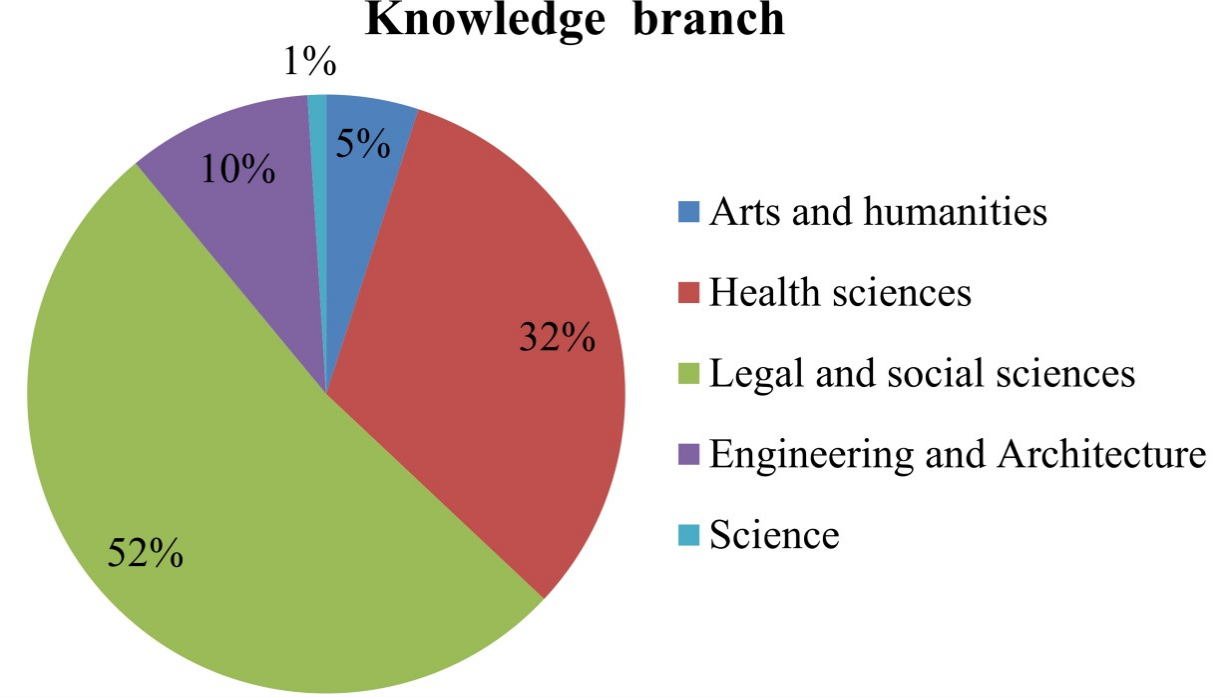
Sample distribution by knowledge branch.

In this study, women have had a greater presence (70.8%) than men (29.2%), and the median age was 21 years old. The vast majority were undergraduate degree students (95.8%, with 4.2% studying master’s degrees). Fig 1 shows the distribution of the sample by knowledge branch.

### Research design and instruments

We designed an *ad hoc* questionnaire based on variables in the models mentioned in the introduction, focusing on those that referred to the didactic process to which we added variables concerning the teacher planning process and methodology. It had a good index of reliability (Cronbach’s alpha = .713)

The questionnaire, titled “Analysis of university student perception of virtual campuses in the EHEA”, comprises fourteen variables of classification and availability of technological resources, and 30 items in six dimensions with responses on a Likert-type scale with four options: 1) Completely disagree; 2) Disagree; 3) Agree; and 4) Completely agree (Table 1).

**Table 1:** Items related to the teaching-learning process in the virtual campus.

Dimensions	Variables	Description
**Teacher planning**	TEPLA1	The competencies and results of learning (objectives) are available on my university’s virtual campus.
	TEPLA2	The content of my subjects are available on my university’s virtual campus.
	TEPLA3	The methodology and work plan for the subjects are available on my university’s virtual campus.
	TEPLA4	Evaluation criteria for the subjects are available on my university’s virtual campus.
**Content**	CONTE1	Subject content on the virtual campus is adapted to students’ prior knowledge.
	CONTE2	Subject content on the virtual campus uses the terminology of the professional discipline.
	CONTE3	Subject content on the virtual campus is up to date.
	CONTE4	Subject content on the virtual campus is created with a variety of didactic elements: diagrams, figures, concept maps, etc.
	CONTE5	Subject content on the virtual campus is available in printable formats (PDF, power point, jpeg, etc.).
	CONTE6	In addition to the main subject content, complementary content in other file formats (text, audio, video, etc.) is published on the virtual campus.
	CONTE7	I access the various files published on the virtual campus (text, audio, video, etc.) as complementary to the main syllabus.
**Methodology**	METHO1	Exercises and practical cases are published on the virtual campus which refer to real problems and situations.
	METHO2	Activities which encourage critical thinking and personal reflection etc. are published on the virtual campus.
	METHO4	Activities which encourage ideas and debate are published on the virtual campus.
	METHO5	Teachers provide advice and guidance in their interactions vial the virtual campus.
	METHO6	Teachers encourage motivation in their interactions through the virtual campus.
**Communication**	COMMU1	Teachers frequently contact me through the virtual campus.
	COMMU2	Communication with teachers through the virtual campus is smooth.
	COMMU3	Teachers respond quickly to questions and observations.
	COMMU4	Teachers respond satisfactorily to questions and observations.
	COMMU5	Teachers ask for evaluations of educational content and techniques in the subject.
**Evaluation**	EVALU1	Before doing a final evaluation, I have self-evaluation exercises available on the virtual campus that are similar to the actual tests.
	EVALU2	In the final grade, a specific percentage of the grade is assigned to participation in online tutorials, forums, chats, etc.
	EVALU3	In the final grade, a specific percentage of the grade is assigned to individual online exercises and activities.
	EVALU4	In the final grade, a specific percentage of the grade is assigned to group exercises and activities.
	EVALU5	I get information from the virtual campus about my grades (messages, individual subject grades etc.)
**Digital competence**	DIGCO1	Teachers have up to date, specialized training to manage the virtual campus.
	DIGCO2	Teachers exhibit a positive attitude towards using the virtual campus.
	DIGCO3	I have appropriate digital competence: knowledge, abilities, skills, values and attitudes related to various technological-digital tools.
	DIGCO4	The use of the virtual campus as a support resource in university teaching-learning gives quality to the education received.

Although there is controversy regarding the optimal number of response alternatives that should make up a Likert-type scale, this type of four-point scale [15] has been chosen in order to eliminate the midpoint, which can increase central tendency and social desirability bias, and allows subsequent dichotomous categorization in a quick and efficient way that facilitates the intuitive reading of the results.

### Procedure

We used an ex post-facto design in this study, focused on the description of an educational phenomenon that had already happened, and on explanatory and predictive analysis of the associations between the different variables [16].

The questionnaire was given to 1048 students meeting the following sample selection criteria: 1) studying at university, preferably undergraduates, and 2) having a virtual campus available in most of their subjects. The instrument was applied by the teachers in the corresponding subjects including it in the virtual campuses.

The procedure followed for this study was carried out in two phases. In the first phase, the different factors relevant to quality assessment in virtual university campuses were studied. For this purpose, a qualitative and quantitative analysis of the study sample based on the previous questionnaire was carried out. Subsequently, decision trees were used to determine the best model for evaluating the quality of education on a virtual campus.

Subsequently, the uncertainty associated with the decision-making processes is introduced into the evaluation model. In order to do this, fuzzy inference systems are used that allow an optimization of the obtained results.

## Results

The following section describes the most relevant results obtained in the two phases of the study. (1) based on decision trees, and (2) based on fuzzy inference systems.

### Model and classification tree

Firstly, as the dependent variable we took the item (DIGCO4): “The use of the virtual campus as a support resource in the university teaching-learning process gives quality to the education received”. This reports the overall evaluation of the educational process, and we understand it as overall satisfaction with the use of the virtual campus in the education process [17].

Following that, and in order to simplify the analysis, as well as to let us analyze the results from two student profiles (those who were satisfied or dissatisfied with the use of the virtual campus as a quality element) we reduced this variable to a dichotomous scale where scores of 1 or 2 on the Likert scale were a negative evaluation, and 3 and 4 were a positive evaluation.

In this way a model was validated (Table 2) which would allow, via classification tree analysis, the determination of the most influential variables.

**Table 2:** Table of model classification.

Risk	
Estimation	Standard Error
**.149**	.011

**Fig 2:**
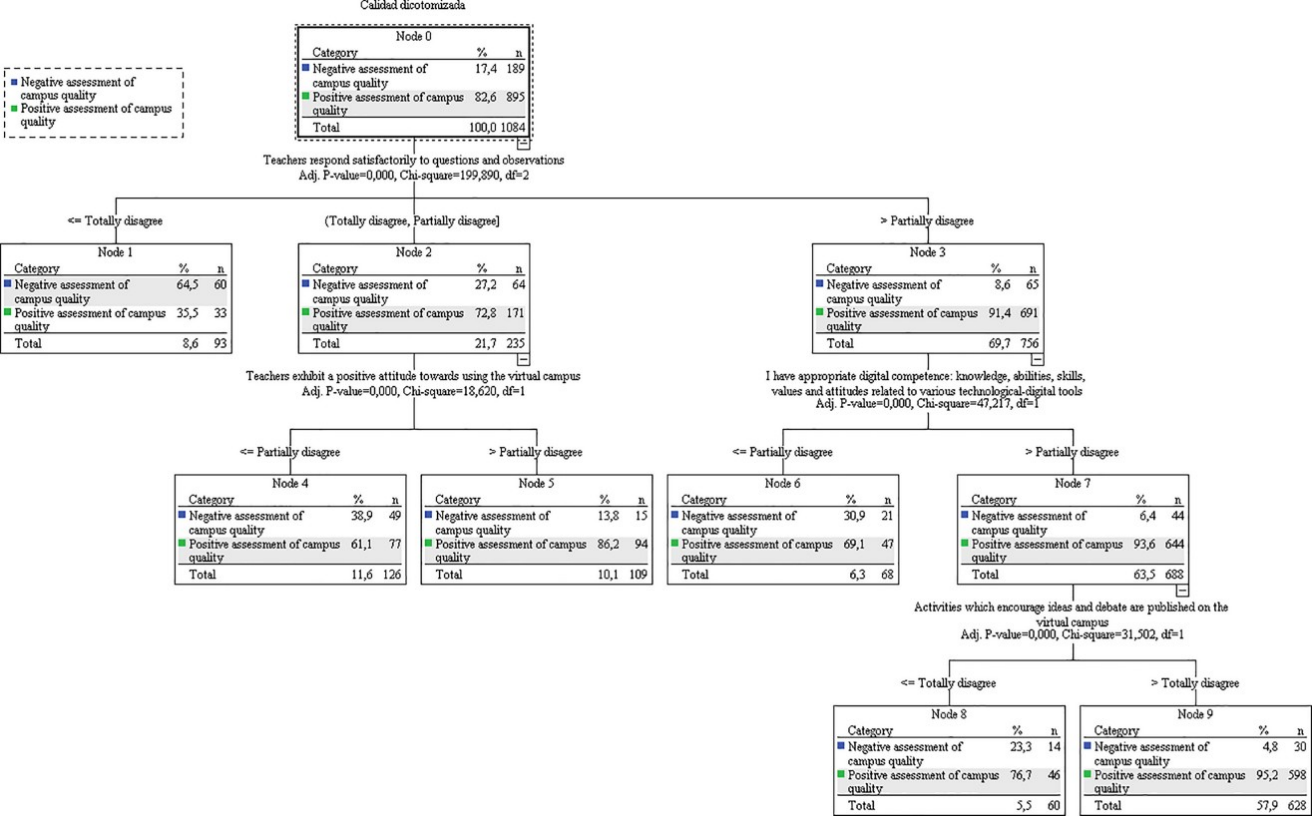
Decision tree referring to the use of the virtual campus as quality criteria in the teaching-learning process.

The profile with a positive evaluation of the campus had high predictive value, with 96.3% of correct assignment. The negative evaluation profile was rejected due to its poor predictive value (31.7%). This is a significant support for those elements making up quality criteria of teaching via virtual campuses which are shown in the decision tree (Fig 2).

As can be seen in the tree, the variable that best predicts a positive evaluation of the campus as a quality element is the satisfactory response of teachers to student questions and observations (χ2 = 199.890; p = .000). If this variable has a positive evaluation (a score of 3 or 4), 91.4% of those subjects have a positive evaluation of the campus. This figure falls to 35.5% in the case of those subjects who completely disagree with the item.

Nonetheless, at a second level, there are two variables that appear to function as modulating elements. The evaluation of a positive teacher attitude towards using the virtual campus (DIGCO2) (χ2 = 18.620; p = .000) to some extent compensates for dissatisfaction with the response to student questions and observations, as those who gave a negative evaluation (≤2) to teacher contact maintain a positive view of campus quality if they evaluate teachers as having a good attitude towards it (86.2%). In addition, within the group that positively evaluated teacher contact (>2) there is a second variable of influence which is self-perception of appropriate digital skills (DIGCO3) (χ^2^ = 47.217; p = .000). Within this group, those who rate their own digital skills as appropriate (>2) tend to ascribe quality to the virtual campus (93.6%).

Something similar happens at a third level with the variable “activities are published on the virtual campus which encourage ideas and debate.” (METHO4) (χ^2^ = 31.502; p = .000), where the majority (95.2%) of those who are not completely negative about this item (>1) are classified in the group that is positive about the educational quality of the virtual campus, provided that this is associated with satisfactory digital communication and self-perception of digital skills.

**Fig 3:**
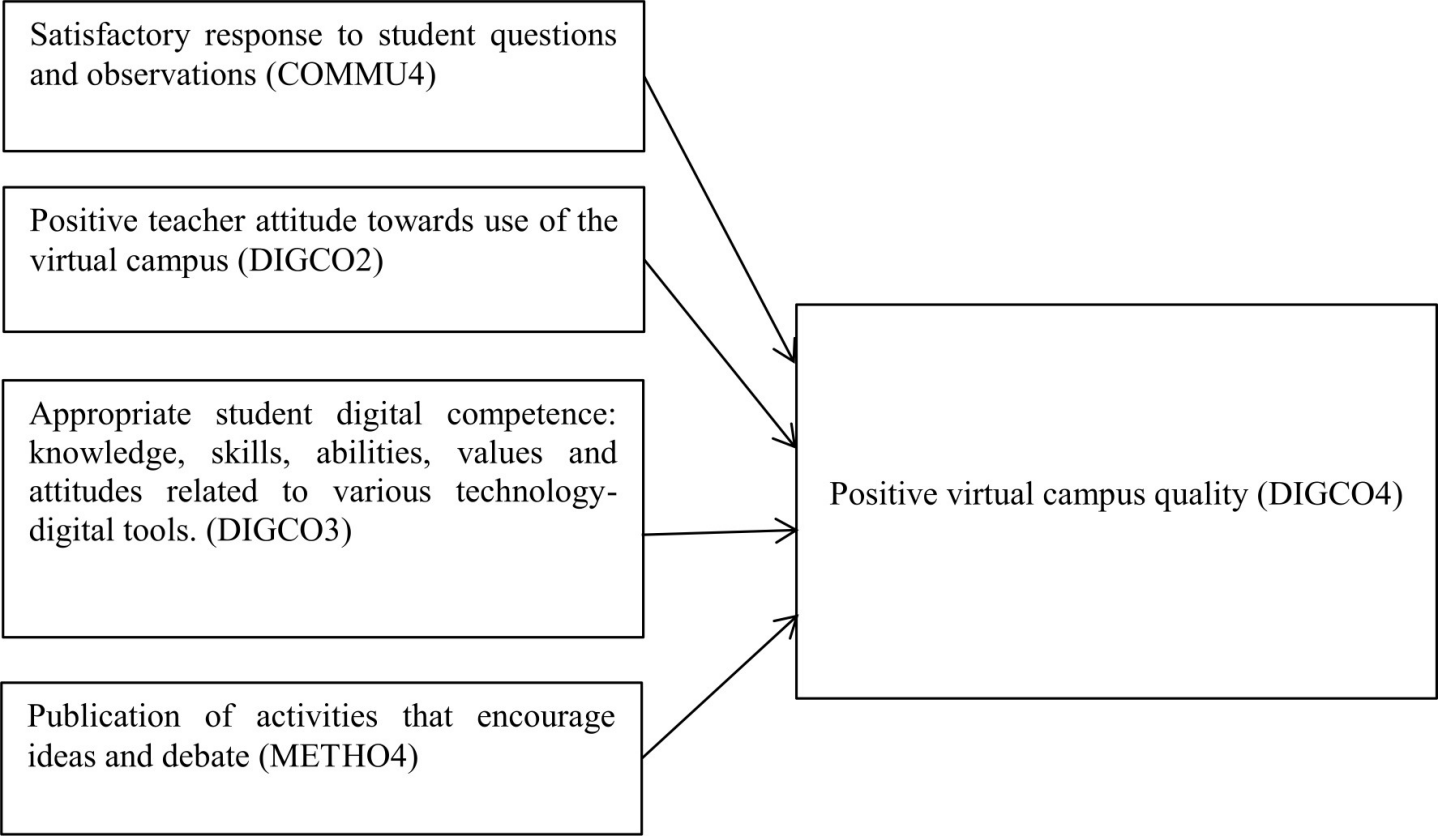
Proposed evaluation model of variables influencing quality of virtual campus.

Following analysis of the decision tree, the model shown in Fig 3 was established for the evaluation of the quality of the virtual educational process.

### Fuzzy inference systems

The use of decision support tools help to achieve more consistent results by implementing expert knowledge in decision making. Those decisions are relaying on subjective assessments, uncertain and therefore difficult to describe accurately and rigorously, even more so if we consider the significant subjectivity inherent in some of the variables in this study [18].

In order to achieve optimum evaluation of the results, we opted to use fuzzy inference systems based on the Fuzzy Sets Theory [19], that allow incorporating the concept of uncertainty to the model, thus making it more robust and efficient. Fuzzy Logic is a strong tool suitable for handling linguistic variables [19], whose values are described in fuzzy numbers. It can be useful for managing the uncertainty associated with different processes such as personal judgement or vague concepts [20]. These systems take place in 5 phases [21, 22]: (1) Fuzzifier -It determines the degree of belonging of each input values to the fuzzy sets. The input value is always a numerical crisp value limited to its domain (in this case [0–1]). Thus, each input value must be fuzzified over all the membership functions associated with the defined labels in the antecedents of each rule -; (2) Application of logical operators–to the fuzzy variables of the antecedents by means of fuzzy relations [23]. The degree of certainty of each rule with its background is determined through the use of logical operators. The most commonly used fuzzy logical operators are: intersection (AND) and union (OR)-; (3) Implication method -it is the one in charge of transferring the fulfillment degree from the antecedent to the consequent of each rule in the evaluation system-; (4) Aggregation of consequences -the consequent of the activated rules are grouped in the final aggregate fuzzy set of each output variable. Thus, a single crisp value can be obtained for each output variable, once the results have been deburred-; (5) Defuzzification -it refers to the method employed to defuzzified the final aggregate, converting it into a crisp value assignable to the output variable by some defuzzification method such as the Centroid of Area, Bisector of Area, Mean of Max, Smallest of Max, and Largest of Max [24]-.

In addition, in these systems, the linguistic type variables allow qualitative and quantitative information to be processed by giving selected labels values related to natural language and common sense reasoning [20, 25, 26] opposite to the traditional numerical variables whose values are exclusively numbers. Fuzzy inference systems are applied in many fields, such as finance, medicine, geology, operation management, among others. In case of the educational quality performance on virtual campuses evaluation, the inherent subjectivity associated to some variables is higher. For this reason, the use of linguistic variables allows a better knowledge management of the evaluation criteria provided by students and experts, while at the same time obtaining more interpretable results capable of gathering all the expert knowledge in the evaluation model. FIS entail the processes of mapping a set of inputs to the output using fuzzy logic. Fuzzy inference mechanism is the fuzzy logic reasoning process that determines the outputs corresponding to fuzzified inputs [23]. The fuzzy rule-base is composed by IF-THEN rules like: IF COMUN4 is low AND DIGCO2 is high AND DIGCO3 is low AND METHOD4 is medium THEN DIGCO4 is low.”

To define this system’s knowledge base, starting from expert technical knowledge, we established the range and form of the fuzzy labels in which the domains of the variables would be divided, and the linguistic rules which, constructed with the items, are able to explain all of the possible evaluations within the proposed evaluation model.

Below, we describe the process of separating the domains and the extraction of knowledge for the rule-base. First, to define the partitions of the variables and the definition of the rule base a panel of 5 subject matter experts was convened to establish the number of labels for the input and output variables of the proposed evaluation model. These labels were defined by considering paired scales, rounding the arithmetic mean from the values assigned by the experts to give 3 labels [low (L), medium (M), high (H)] for the input variables, and 5 labels for the final evaluation [very low (MB), low (L), medium (M), high (H), very high (VH)]. The semantic representation of the labels was associated with the trapezoidal fuzzy numbers, as they were sufficiently robust to represent the vagueness of the linguistic evaluations provided by primary and secondary research sources of information [27]. In addition, this type of fuzzy partition demonstrates better results in terms of understanding and satisfaction of significant semantic restrictions, such as distinction, normalization, coverage and superposition.

**Fig 4:**
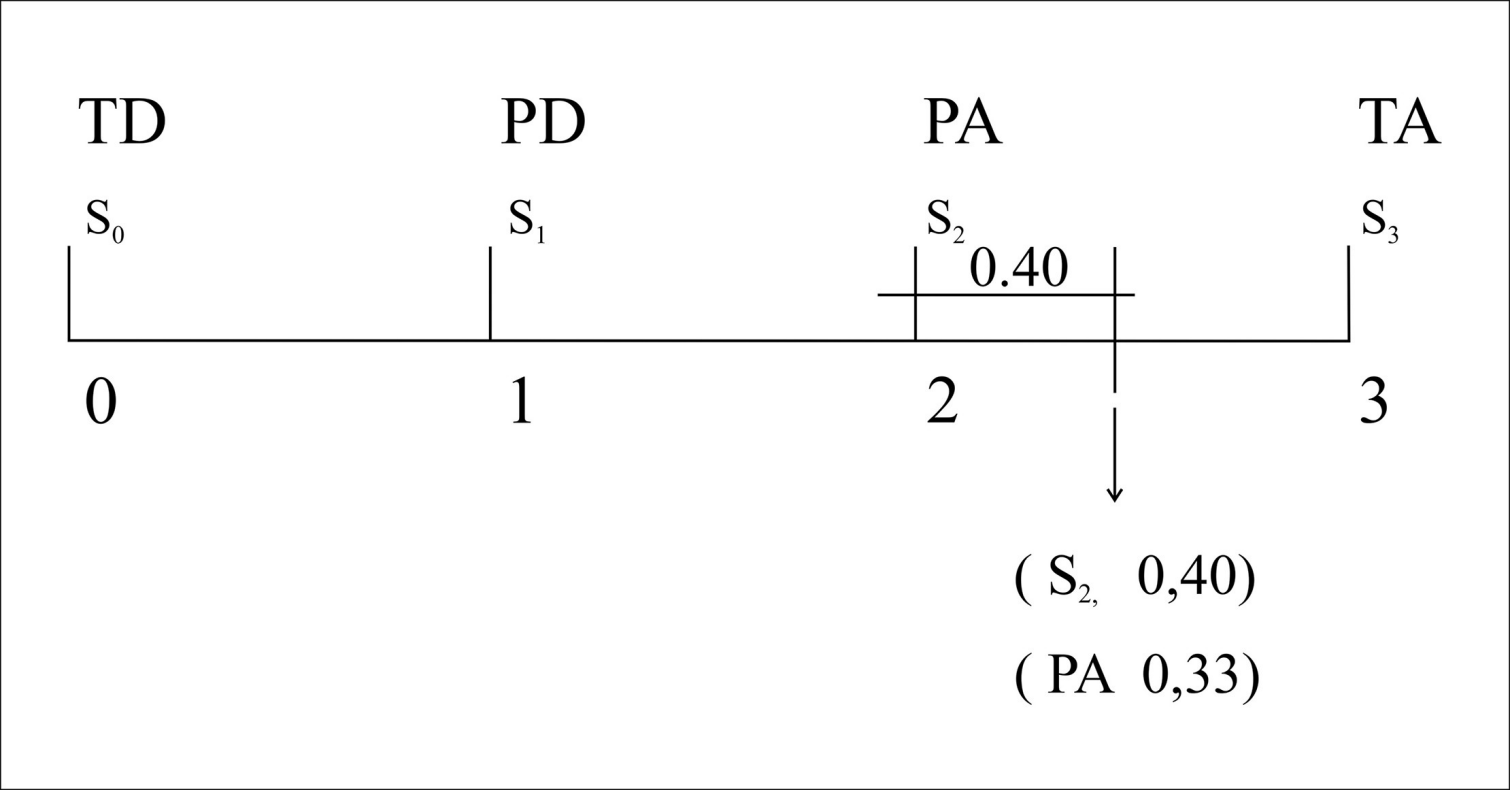
Alternative core-with structures.

In order to partitions the variable to defined the domains in the inference subsystems of the model, we used a 2-tuple fuzzy linguistic representation model based on a symbolic translation that would allow the representation and management of linguistic information through the value pairs s_i_ and α_i_, where “s_i_” is a linguistic term from an original set “S” of linguistic terms, and “α_i_” is a numerical value evaluated in the interval [-0.5, 0.5) [28]. In order to do that, we defined a set of linguistic terms of preference (Table 3) so that the experts could evaluate the different core-width structures of the labels agreed by the experts for each variable in the proposed model as we can see in Fig 4.

**Table 3:** Scale of linguistic preferences comparable to each alternative structure.

	Level	Concept	Trapezoidal fuzzy numbers	2-Tuples
S_0_	TD	Totally disagree	(.0 .0 .2 .4)	(TD, 0)
S_1_	PD	Partially disagree	(.2 .4 .5 .7)	(PD, 0)
S_2_	PA	Partially agree	(.5 .7 .7 .9)	(PA, 0)
S_3_	TA	Totally agree	(.7 .9 1.0 1.0)	(TA, 0)

The experts provided the preference evaluations illustrated in Table 4 with regard to these four possible structures.

**Table 4:** Evaluations of linguistic preference provided by experts in 4 alternative structures to define the partition cores.

	Estruc_1	Estruc_2	Estruc_3	Estruc_4
**Expert 1**	TD	TA	PD	PA
**Expert 2**	TA	PD	TA	TD
**Expert 3**	PD	TA	TD	PA
**Expert 4**	TD	PA	PD	TA
**Expert 5**	TD	TA	PD	PA
**Extended arithmetic mean**	.20	2.40	1.20	1.80
**2-Tuples:**	(TD, .20)	(PA, .40)	(PD, .20)	(PA, -.20)

**Fig 5:**
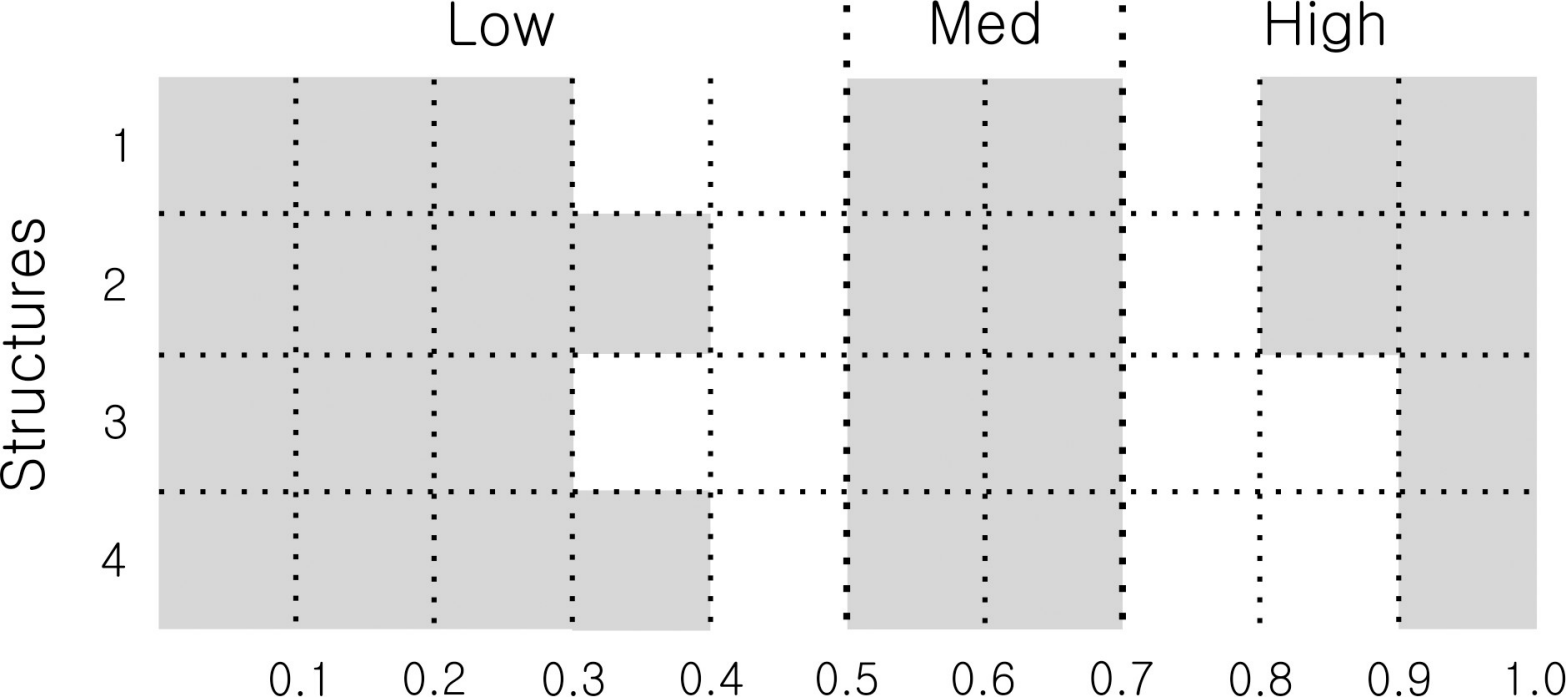
Symbolic translation of extended arithmetic mean = 2.40 to 2-tuple = (PA. 0.40).

As a means of aggregation for the combined evaluation of each structure, we used the extended arithmetic mean (EAM) based on the order of the labels in their scales. From these values 2-tuples associated with each structure were configured, one of which allowed identification of the aggregated preferences, and through a process of symbolic translation based on the interval [-0.5–0.5) [29], the closeness to the left or right (Fig 5). Once the 2-tuples of all of the alternative structures were identified, the one which represented the best aggregate preference was chosen, in this case structure two.

**Fig 6:**
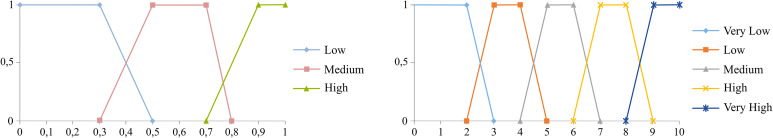
Partition of the domain of input (left) and output (right) variables of the evaluation model.

Finally, the semantics of each label were developed, via fuzzy trapezoidal numbers with the criteria established to achieve a strong partition in each variable. This followed an identical procedure to that described in the output variables of the model of quality evaluation in the virtual campus (Fig 6).

We conclude this description of the statistical analysis referring to the method of elicitation based on the principle of extension [30] as a procedure of diffusion of the rule-base. The experts were asked to choose, for the fuzzy inference system of the proposed model, the preferred output labels in each combination of input labels, according to the previously defined partitions. The evaluations from the experts were put together with the input variables from the inference model.

With these evaluations, using the arithmetic mean aggregation method, following the principle of extension, we obtained the “collective preference vectors” associated with the combination of input variable labels of the system being analyzed.

Similarly, to fit these preference vectors to one of the five labels corresponding to the partition of the model, the distance of each vector from the five labels was calculated using the following formula for distance, taking into account the five partition labels of the output variable {very low (MB), low (B), medium (M), high (A) and very high (MA)} and taking Pa = Pd = 0.15 and Pb = Pc = 0–35.

$$D(\left[a_{i},b_{i},c_{i},d_{i}]-[a^{j},b^{j},c^{j},d^{j}\right])=\sqrt{P_{a}{\left(a_{i}-a^{j}\right)}^{2}+P_{b}{\left(b_{i}-b^{j}\right)}^{2}+P_{c}{\left(c_{i}-c^{j}\right)}^{2}+P_{d}{\left(d_{i}-d^{j}\right)}^{2}}$$ (1)

In this case [a_i_,b_i_,c_i_,d_i_] represents the i^th^ preference vector, [a^j^,b^j^,c^j^,d^j^] represents the j^th^ label of the original partition and Pa, Pb, Pc and Pd are the significances of the weights associated with the fuzzy numbers considered.

For the 36 preference vectors, the minimum distances in each row were obtained, thus identifying the final output label to assign in each of the 81 model combinations. To summarize, Table 5 shows an example of analysis for 7 of the 81 possible combinations.

**Table 5:** Collective preference vectors of input variables and label assignment in the evaluation model rule-base.

COMMU4	DIGCO2	DIGCO3	METHO4	Collective preference vectors				Distances					Output variable labels
								D(VL)	D(L)	D(M)	D(H)	D(VH)	
**L**	M	L	L	.00	.00	2.20	4.02	.00	2.20	4.02	5.85	7.70	**VL**
**M**	H	M	L	2.40	2.61	.39	1.47	2.61	.39	1.47	3.32	5.17	**L**
**H**	L	M	L	2.40	2.70	.44	1.39	2.70	.44	1.39	3.24	5.10	**L**
**H**	L	M	M	4.40	4.53	2.29	.44	4.53	2.29	.44	1.39	3.26	**M**
**H**	M	H	L	3.60	3.80	1.55	.28	3.80	1.55	.28	2.13	3.99	**M**
**H**	M	H	M	5.60	5.64	3.40	1.55	5.64	3.40	1.55	.28	2.16	**H**
**H**	M	H	H	7.60	7.47	5.23	3.38	7.47	5.23	3.38	1.52	.33	**VH**

Once the rule base was defined, it was added to the fuzzy inference system using the Matlab 6.5 “Fuzzy” toolbox, inferring the evaluation of positive quality of the virtual campus according to the “crisp” values assigned to the input variables. It is simple and intuitive to analyze the congruence of the evaluations via inference maps provided for the current model. In these maps, the evaluations are given by the height over a surface at each point, while for the analysis of our results it was necessary to perform the evaluation in terms of the two input variables for a constant value of the third variable not shown in the graphic.

**Fig 7:**
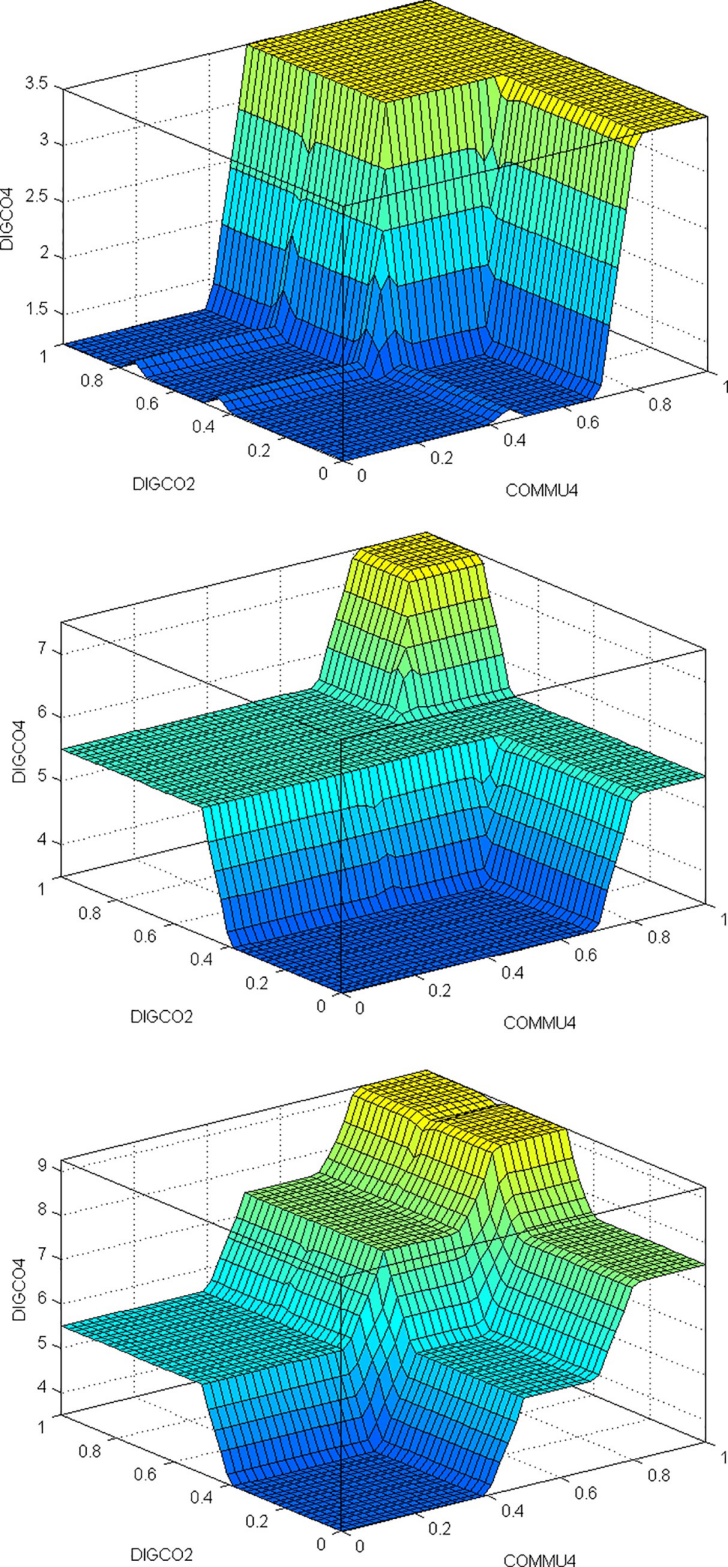
Solution maps of DIGCO4 evaluation, with DIGCO3 and METHO4 low (left) medium (center) and high (right).

One possible option is to reflect the final evaluation of the quality of the virtual campus (DIGCO4) against the variables of satisfactory response to questions and observations (COMMU4) and positive attitude of teachers towards using the virtual campus (DIGCO2) for different values of the students’ perceptions of their own technological skills (DIGCO3) and the publication of activities that encourage ideas and debate (METHO4) (Fig 7).

In Fig 7 we see that for values of communication (COMMU4) or positive attitudes towards the virtual campus (DIGCO2) below .4, the output is zero (regardless of the other variables) as set out in the rule-base. Nonetheless, as the values of these variables increase, so does the positive quality of the virtual campus (DIGCO4), and with it, student satisfaction with the campus. This is logical, satisfactory communication with teachers, through the domain of digital tools, and the quality of these tools through appropriate instructional design, may compensate to some extent for negative values in the positive attitude of teachers about the use of more active methodologies.

We also see that for medium and high values in students’ perceptions of their own digital competence (DIGCO3) and the publication of activities encouraging ideas and debate (METHO4), high output areas are reached for a broad region of the solution map, and as such, higher student scores about the quality of the virtual campus. The synergetic effect of the variables increases. They reinforce the communicative, digital and didactic or methodological side of the teaching-learning process, which impacts in turn on the overall quality of the educational process.

**Fig 8:**
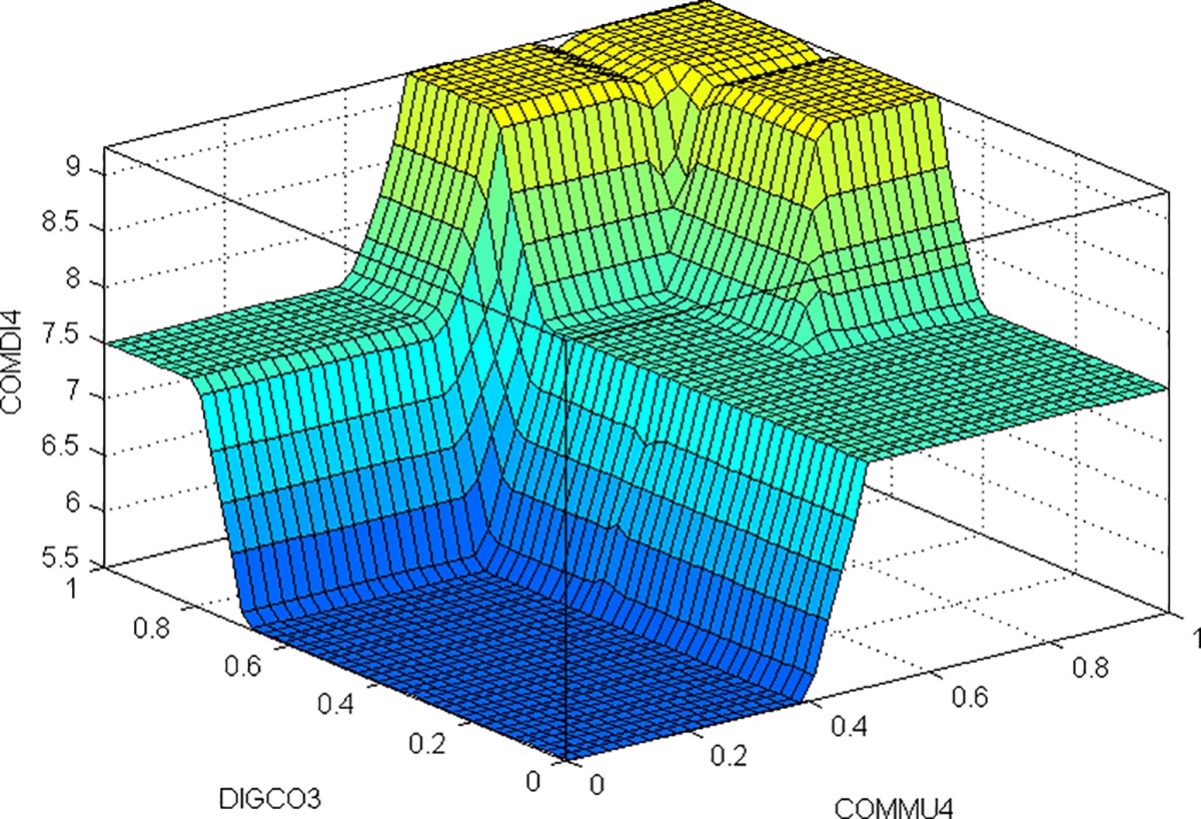
Solution map of DIGCO4 evaluation, with COMMU4 and METHO4 high.

Another example of analysis is to look at the change in virtual campus quality based on high values in teachers communicative skills (COMMU4) and the active methodological strategies encouraging ideas and debate (METHO4) for different values in the variables of positive attitude towards the use of the campus (DIGCO2) and student digital competence (DIGCO3) (Fig 8).

From this figure we see that for medium perceptions of student digital competence (DIGCO3) with scores over .4 points, the results in virtual campus quality are higher for a broader area of the solution map. Similarly, the score for the positive attitude of teachers towards the use of the virtual campus increases (DIGCO2), rising from 5.5 points for low scores to 7.5 or 9 points for medium and high scores respectively. This is because students with better digital skills perceive the virtual campus as having higher quality, and this has a positive influence on its relationships with the other variables in the model. In addition, we see that for scores below 0.4 points in student digital skills, and .7 in teachers’ attitudes towards the use of the virtual campus, the evaluation of the quality of the virtual campus is zero, regardless of the methodological or communication variables. This shows that in order to have a good evaluation of the management of the virtual campus, the teachers or the university need to ensure that students have good perceptions of the variables mentioned.

## Discussion and conclusions

The model created from the classification tree indicates four particularly important variables that might guide good teaching practice and lead to the establishment of an optimal educational model for virtual campuses: 1) satisfactory teacher responses to student questions and observations (COMMU4), in the communicative dimension; 2) a positive attitude from the teachers about using the virtual campus (DIGCO2) and 3) students having appropriate digital skills (DIGCO3), in the digital competence dimension; and 4) publication of activities that encourage ideas and debate (METHO4), in the dimension referring to methodological or didactic content.

In addition to that, we selected a fuzzy inference system that provides greater possibilities of intuitive interpretation of the evaluation of the virtual campus by exhibiting better interpretation of the information in the evaluation decision thanks to fuzzy logic.

With that in mind, we can draw some conclusions in line with previous research that underline the importance of the variables found in educational practice with the use of ITC. Firstly, communicative feedback between teachers and students, including good relationships with students, has been repeatedly highlighted in the scientific literature. Authors such as [28] have underlined the importance of using technology tools with communicative ends to improve tutoring, and [31] agreed that a smooth relationship with teachers reduces the likelihood of students dropping out.

Secondly, it seems obvious that a positive attitude of teachers towards the use of ITC [32] as well as previous training for students in its use [33] should result in an increase in positive perceptions of it as an educational method [25–34,35]. We agree with [36], who identified a positive relationship between digital skills, attitude towards ITC and the virtual learning environment, which is why many educational establishments and universities are already integrating digital skill development into their teaching plans [37].

Finally, there is a need to improve the methodological side through the inclusion of activities that encourage ideas and debate. This is particularly important when we think that one of the characteristics of the knowledge society is information overload. The challenge is not in access to information, but rather in discussing and comparing ideas, weighing the arguments of different valid alternatives in order to lay down significant knowledge in continual progression. Our results are in line with [38], who indicated that students were more proactive, and had better achievement when methodologies were used which focused on participation and debate, and with [39], who reported the didactic potential of strategies based on collaborative learning that demand confrontation of ideas, such as the “flipped classroom” model, MOOCs, individualized learning environments, game-based learning, and learning analyses.

In terms of implications, our results about the evaluation of virtual campuses may help universities identify the strengths and weaknesses in their online teaching-learning processes. This may allow them, as [8] and [28] anticipated, to adopt preventive and corrective measures to improve future evaluations. Furthermore, if these institutions can dynamically collect and process student perceptions about the variables in the model, they may be able to better monitor how evaluations change over time, including in how they compare with other universities. That will not only improve the quality of their own educational processes, based on the construction of personal learning networks [40,41], but also in their overall reputation and image. The main limitation of the research concerns the sample used, which should be extended in future studies in order to increase the external validity of the research. In addition, statistically significant differences have been found between men and women, being especially relevant for our work those existing in the variables of perception of digital competence itself and the assessment of lecturers' attitudes towards the virtual campus. This aspect, together with the existing differences by university ownership, for which it will be necessary to increase the sample of students from private universities, are foreseen as future lines of research. Additionally, it would be interesting to increase the sample size and broaden the study to other European universities in order to compare the strengths and weaknesses of their respective virtual campuses, drawing on perception-positioning maps and then introducing methods based on artificial intelligence such as Fuzzy-AHP or Fuzzy-TOPSIS, with the aim of looking again at the uncertainty associated with multi-criteria decision processes. The research could conclude with the incorporation of intelligent systems, able to predict and adapt the proposed model depending on student perceptions.

## References

[1] Pozos KV, Mas O. The digital competence as a cross-cutting axis of higher education teachers’ pedagogical competences in the European higher education area. Procedia Soc Behav Sci. 2012;46:1112–16. 10.1016/j.sbspro.2012.05.257

[2] Bengoetxea E, Buela-Casal G. The new multidimensional and user-driven higher education ranking concept of the European Union. Int J Clin Health Psychol. 2013;13(1): 67–73. 10.1016/S1697-2600(13)70009-7

[3] Zineldin M, Akdag HC, Vasicheva V. Assessing quality in higher education: New criteria for evaluating students’ satisfaction. Quality in Higher Education. 2011;17(2):231–43. 10.1080/13538322.2011.582796

[4] Tarhini A, Hone K, Liu X. User acceptance towards web-based learning systems: Investigating the role of social, organizational and individual factors in European higher education. Procedia Comput Sci. 2013;17:189–97. 10.1016/j.procs.2013.05.026

[5] Chichernea V. Campus information systems for enhancing quality and performance in a smart city high education environment. Conference Proceedings of eLearning and Software for Education (eLSE) (No. 01, pp. 50–56). “Carol I” National Defence University Publishing House. Available from https://bit.ly/2PXCdRn (2016).

[6] Stefan L, Moldoveanu F, Gheorghiu D. Evaluating a Mixed-Reality 3D Virtual Campus with Big Data and Learning Analytics: A transversal study. Journal of e-Learning and Knowledge Society. 2016;12(2), Italian e-Learning Association. Retrieved November 12, 2019 from https://bit.ly/2r6Z18V

[7] Wastiau P, Blamire R, Kearney C, Quittre V, Van de Gaer E, Monseur C. The use of ICT in Education: A Survey of Schools in Europe. Eur J Educ. 2013;48(1):11–27. 10.1111/ejed.12020

[8] Benson V, Morgan S. Social university challenge: Constructing pragmatic graduate competencies for social networking. Br J Educ Technol. 2016;47(3):465–73. 10.1111/bjet.12448

[9] Tarling I, Ng'ambi D. Teachers pedagogical change framework: a diagnostic tool for changing teachers’ uses of emerging technologies. Br J Educ Technol. 2016;47(3):554–72. 10.1111/bjet.12454

[10] Ossiannilsson E, Landgren L. Quality in e-learning a conceptual framework based on experiences from three international benchmarking projects. J Comput Assist Learn. 2012;28(1):42–51. 10.1111/j.1365-2729.2011.00439.x

[11] Sarrab M, Elbasir M, Alnaeli S. Towards a quality model of technical aspects for mobile learning services: An empirical investigation. Comput Human Behav. 2016;55:100–12. 10.1016/j.chb.2015.09.003

[12] Andone D, Ternauciuc A, Vasiu R. Using Open Education Tools for a Higher Education Virtual Campus. In: Chang M, Chen NS, Huang R, Kinshuk, Sampson DG, Vasiu, R, editors. IEEE 17th International Conference on Advanced Learning Technologies (ICALT); 2017 Jul 3–7; Timisoara, Romania. California: IEES Computer Society and Conference Publishing Services; 2017. p. 26–30. Available from https://bit.ly/2M8Sq57

[13] Martos-García D, Usabiaga O, Valencia-Peris A. Students’ Perception on Formative and Shared Assessment: Connecting two Universities through the Blogosphere. Journal of New Approaches in Educational Research. 2017; 6(1): 64–70. 10.7821/naer.2017.1.194

[14] Deb S, Bhattacharya PA. Framework to Enhance the Learning Outcome with Fuzzy Logic-Based ABLS (Adaptive Behaviourial Learning System). In: Saeed K, Chaki N, Pati B, Bakshi S, Mohapatra DP(editors.). Progress in Advanced Computing and Intelligent Engineering Singapore: Springer; 2018 p.3–11.

[15] Nadler JT, Weston R, Boyles EC. Stuck in the middle: the use and interpretation of mid-points in items on questionnaires. The Journal of General Psychology. 2015; 142(2):71–89. 10.1080/00221309.2014.994590 25832738

[16] Derakhshanrad SA, Piven EA. Cognitive Neurodynamic Approach to Prediction of Students’ Adaptation to College: An Ex-Post Facto Study. Basic Clin Neurosci. 2018;9(3): 217–26. 10.29252/nirp.bcn.9.3.217 30034652 PMC6037428

[17] Hernández-Nanclares N, Pérez-Rodrígez M. Students' Satisfaction with a Blended Instructional Design: The Potential of "Flipped Classroom" in Higher Education. Journal of Interactive Media in Education. 2016;(1):4 10.5334/jime.397

[18] Maio GR, Haddock G, Verplanken B. (2018). The psychology of attitudes and attitude change. Sage Publications Limited Available from https://bit.ly/2CB8LdO

[19] Zadeh LA. Fuzzy Sets. Information and Control. 1965; 8(3): 338–53.

[20] Centobelli P, Cerchione R, Esposito E. Efficiency and Effectiveness of Knowledge Management Systems in SMEs, Product. Plan & Cont. 2019; 30(9):779–91. 10.1080/09537287.2019.1582818

[21] Ross TJ. Fuzzy logic with engineering applications. John Wiley & Sons, Ltd New Mexico, USA; 2010.

[22] Blej M, Azizi M. Comparison of Mamdani-Type and Sugeno-Type Fuzzy Inference Systems for Fuzzy Real Time Scheduling. International Journal of Applied Engineering Research. 2016; 11(22):11071–75.

[23] Blej M, Azizi M. Comparison of Mamdani-Type and Sugeno-Type Fuzzy Inference Systems for Fuzzy Real Time Scheduling. International Journal of Applied Engineering Research. 2016; 11(22):11071–75.

[24] Carvallaro F. A Takagi-Sugeno Fuzzy Inference System for Developing a Sustainability Index of Biomass. Sustainability. 2015;7(9): 12359–71; 10.3390/su70912359

[25] Watanabe. Statistical Methods for Estimating Membership Functions. Japanese Journal of Fuzzy Theory and Systems, (1979), 5, 4.

[26] Khuman A, Yang Y, John R. The quantification of subjectivity: The R-fuzzy grey analysis framework. Expert Syst Apply. 2019; 131:201–16. 10.1016/j.eswa.2019.06.043

[27] Mahmoum-Gonbadi A, Katebi Y, Doniavi A. Generic two-stage fuzzy inference system for dynamic prioritization of customers. Expert Syst Appl. 2019;131:240–53. 10.1016/j.eswa.2019.04.059

[28] Fernández A, Del Jesús MJ, Herrera F. On the 2-tuples based genetic tuning performance for fuzzy rule based classification systems in imbalanced data-sets. Inf Sci. 2010;180(8):1268–91. 10.1016/j.ins.2009.12.014

[29] Shojaiemehr B, Rafsanjani MK. A supplier offer modification approach based on Fuzzy systems for automated negotiation in e-commerce. Inf Syst Front. 2018;20(1):143–60.

[30] Mencar C, Fanelli AM. Interpretability constraints for fuzzy information granulation, Inf Sci. 2008;178:4585–4618. 10.1016/j.ins.2008.08.015

[31] Dubois D, Prade H. Systems of linear fuzzy constraints. Fuzzy Sets Syst. 1980;3(1):37–48.

[32] Scott K. Does a university teacher need to change e-learning beliefs and practices when using a social networking site? A longitudinal case study. Br J Educ Technol. 2013;44(4):571–80. 10.1111/bjet.12072

[33] Bernardo A, Esteban M, Fernández E, Cervero A, Tuero E, Solano P. Comparison of personal, social and academic variables related to University Dropout and Persistence. Front Psychol. 2016;7:1610 10.3389/fpsyg.2016.01610 27803684 PMC5067534

[34] Rojano S, López MM. Teaching sciences using didactic strategies based in information and communications technologies. Advances in Higher Education. 2017;1(2):1–9. Available from https://bit.ly/2PX4VBO

[35] Krumsvik RJ. Teacher educators' digital competence. Scandinavian Journal of Educational Research. 2014;58(3):269–80. 10.1080/00313831.2012.726273

[36] Área M, Hernández VM, Sosa JJ. Modelos de integración didáctica de las TIC en el aula. Comunicar, 2016;24(47):79–87. 10.3916/C47-2016-08

[37] Cerezo R. et al Learning difficulties in Computer-Based Learning Environments. In: González-Pienda JA, Bernardo A, Núñez JC, Rodríguez C, editors. Factors Affecting academic performance. New York: Nova Science; 2017 p. 157–70.

[38] Trevors G, Feyzi-Behnagh R, Azevedo R, Bouchet F. Self-regulated learning processes vary as a function of epistemic beliefs and contexts: Mixed method evidence from eye tracking and concurrent and retrospective reports. Learn Instr, 2016;42:31–46. 10.1016/j.learninstruc.2015.11.003

[39] Colon AO, Galiano IM, Colmenero-Ruiz M. Impact of the Flipped Classroom Model and Collaborative Learning in Childhood Teaching University Degree. *Journal of e-Learning and Knowledge Society*, 2017,13(3). Available from: https://bit.ly/38X5ch1

[40] Hatlevik OE, Christophersen KA. Digital competence at the beginning of upper secondary school: identifying factors explaining digital inclusion. Comput Educ. 2013;63:240–7. 10.1016/j.compedu.2012.11.015

[41] Corell A, Regueras LM, Verdú E, Verdú MJ, de Castro JP. Effects of competitive learning tools on medical students: A case study. *PloS ONE*. 2018;13(3). 10.1371/journal.pone.0194096 29518123 PMC5843339

